# First molecular-based detection of SARS-CoV-2 virus in the field-collected houseflies

**DOI:** 10.1038/s41598-021-93439-7

**Published:** 2021-07-06

**Authors:** Aboozar Soltani, Marzieh Jamalidoust, Amin Hosseinpour, Mozaffar Vahedi, Hadi Ashraf, Saeed Yousefinejad

**Affiliations:** 1 Research Center for Health Sciences, Institute of Health, Department of Medical Entomology and Vector Control, School of Health, Shiraz University of Medical Sciences, Shiraz, Iran; 2 Clinical Microbiology Research Center, Shiraz University of Medical Sciences, Shiraz, Iran; 3 Department of Medical Entomology and Vector Control, School of Health, Shiraz University of Medical Sciences, Shiraz, Iran; 4 Communicable Disease Unit, Shiraz University of Medical Sciences, Shiraz, Iran; 5 Research Center for Health Sciences, Institute of Health, School of Health, Shiraz University of Medical Sciences, Shiraz, Iran

**Keywords:** Microbiology, Molecular biology, Zoology, Diseases, Pathogenesis

## Abstract

This is the first report of SARS-CoV-2 detection on field-collected *Musca domestica* housefly surface and tissue samples using the high-sensitive PCR assay which suggests the possible insect-borne transmission. The study was conducted in Shiraz city, southern Iran, in May and Jun 2020. Adult flies were sampled at the outdoor areas of two hospitals treating COVID-19 patients. Fly samples were first washed twice to remove the insect surface attached to SARS-CoV-2 virions. After that, the disinfected fly samples were homogenized. Fly surface washout and homogenate samples were tested using Taq Man real-time PCR assay for the SARS-CoV-2 virus. In a total of 156 houseflies, 75% of samples from the body washout samples were positive for SARS-CoV-2. Strikingly, 37% of the homogenized specimens were positive for the SARS-CoV-2, suggesting the possible infection of the insects or uptake of the virion to the insect metabolism. The other possibility is the houseflies up took the blood or blood fluids of the patients and the RNA of the SARS-CoV-2 survived in the insect body without replicating. Our preliminary findings suggest that the houseflies could transmit SARS-CoV-2 as a mechanical or biological vector especially during the warm seasons while increasing the population and activity of houseflies.

## Introduction

Coronavirus disease 2019 (COVID-19) is an acute respiratory illness reported from Wuhan City, Hubei Province, China in late 2019^1^.

Two main mechanisms for SARS-CoV-2 virus transmission have been introduced, including transmission through air droplets and fomites^2,3^. The delivery of the viruses with the houseflies could be a new way to add to the previously described ways by researchers.

There are various studies in the world on the transmission of pathogens by insects. Biological and mechanical transmission methods are the two main reasons for the transmission of communicable diseases or epidermis. The possibility of transmitting viral diseases that cause acute respiratory infections in humans and animals has been studied in different parts of the world and the possibility of mechanical transmission of these viruses by some species of insects has been proven^4–14^.

The housefly (*Musca domestica*) is a non-blood feeding insect of the order Diptera that can be a mechanical vector of many diseases, especially nosocomial infections, due to its unique behavioral and nutritional mechanisms. This insect can transmit pathogens mechanically to humans in four main ways: (1) adherence of pathogens to its body through the legs, wings, and surface of the abdomen, etc. (2) By its diverse diet, which is omnivorous and can feed on anything such as household and hospital waste, mucus secretions such as sputum, as well as feces, etc. (3) Regurgitation behavior on foods, and (4) defecation behavior on surfaces and foods. The combination of these behaviors causes the mechanical transmission of many pathogens^15^. We also know from previous studies that, the housefly might suck the liquids such as sweat, or fecal that contains the SARS-CoV-2 and the insect might contain the RNA only.

Also, it has been proven that the SARS-CoV-2 could enter the host cells using cell surface components^16^. Some of these components have existed in the insects^16^. SARS-CoV-2 spike could hook up the insect body using these receptors^17^.

Given that Covid-19 is a new, deadly, and highly contagious disease worldwide, most countries are now more focused on preventing, controlling, and treating patients. Therefore, many different epidemiological aspects of this new virus and its transmission mechanisms are unknown to humans.

Some countries affected by the disease face with increasing temperature in the warm seasons of the year, leading to an increase in the population of houseflies and other synanthropic flies. If these insects can enter the disease transmission cycle and play a role in the mechanical transmission of the disease, it will make the disease control operation very difficult and the health system will face a very serious challenge^18^.

Therefore, in this study, we surveyed the existence of the SARS-CoV-2 in the field-collected houseflies by the identification molecular method for the first time in the world. Such investigations are very important because of the high abundance of houseflies and the possibility of its effect on infection transmission.

## Methods

### Study area

**Figure 1:**
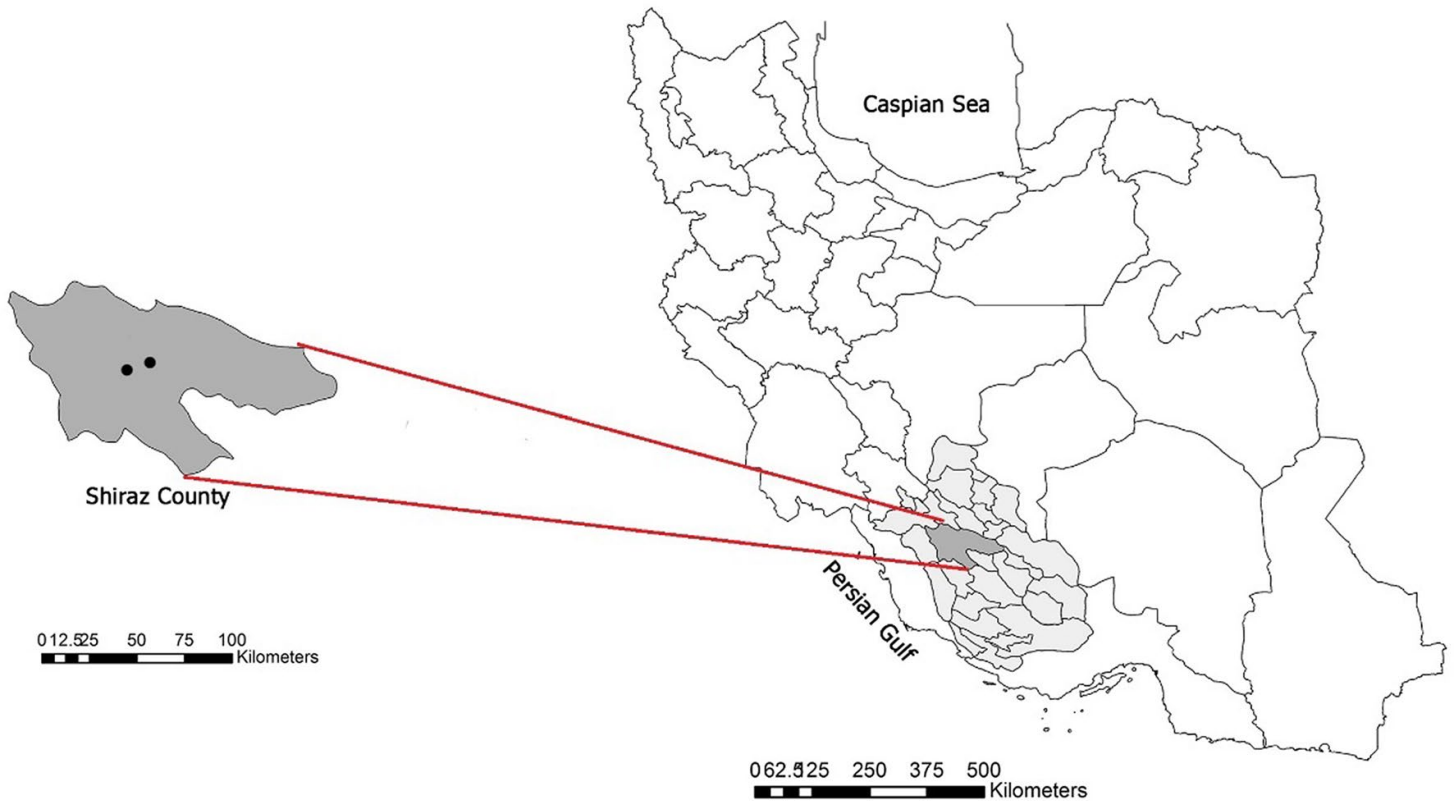
Map of Shiraz county (the capital of Fars province, south of Iran) showing the sampling locations (generated using ArcGIS Desktop 10.8 software).

This cross‐sectional study conducted in Shiraz (29° 40′ N, 52° 33′ E), the capital city of Fars province, southern Iran. It is located at about 1500 m above sea level. It has a subtropical, hot, semi‐arid climate with a mean annual temperature of 18 °C, relative humidity of 41%, and precipitation of 337.8 mm. Its hilly landscape is corrugated with the Zagros Mountain range, which runs northwest-southeast across the country. Two main hospitals (Ali-Asghar hospital with coordinates 29.625959 N 52.541238 E and Chamran hospital with coordinates 29.662890 N 52.490799 E) were the spot point of this work which were two main referral centers of COVID-19 treatment in Shiraz (Fig. 1). Both of these referral hospitals are located in the center of the city with a clean environment and without any abnormal source of pollution, accumulation of garbage, or sewage around its location.

### Collection and identification of flies

In this study, in coordination with the health department of Shiraz University of Medical Sciences, Muscid flies sampling operations were performed from outdoor environments of the two target hospitals (Ali-Asghar hospital and Chamran hospital) dealing with Covid-19 patients for 8 weeks from May to July 2020.

**Figure 2:**
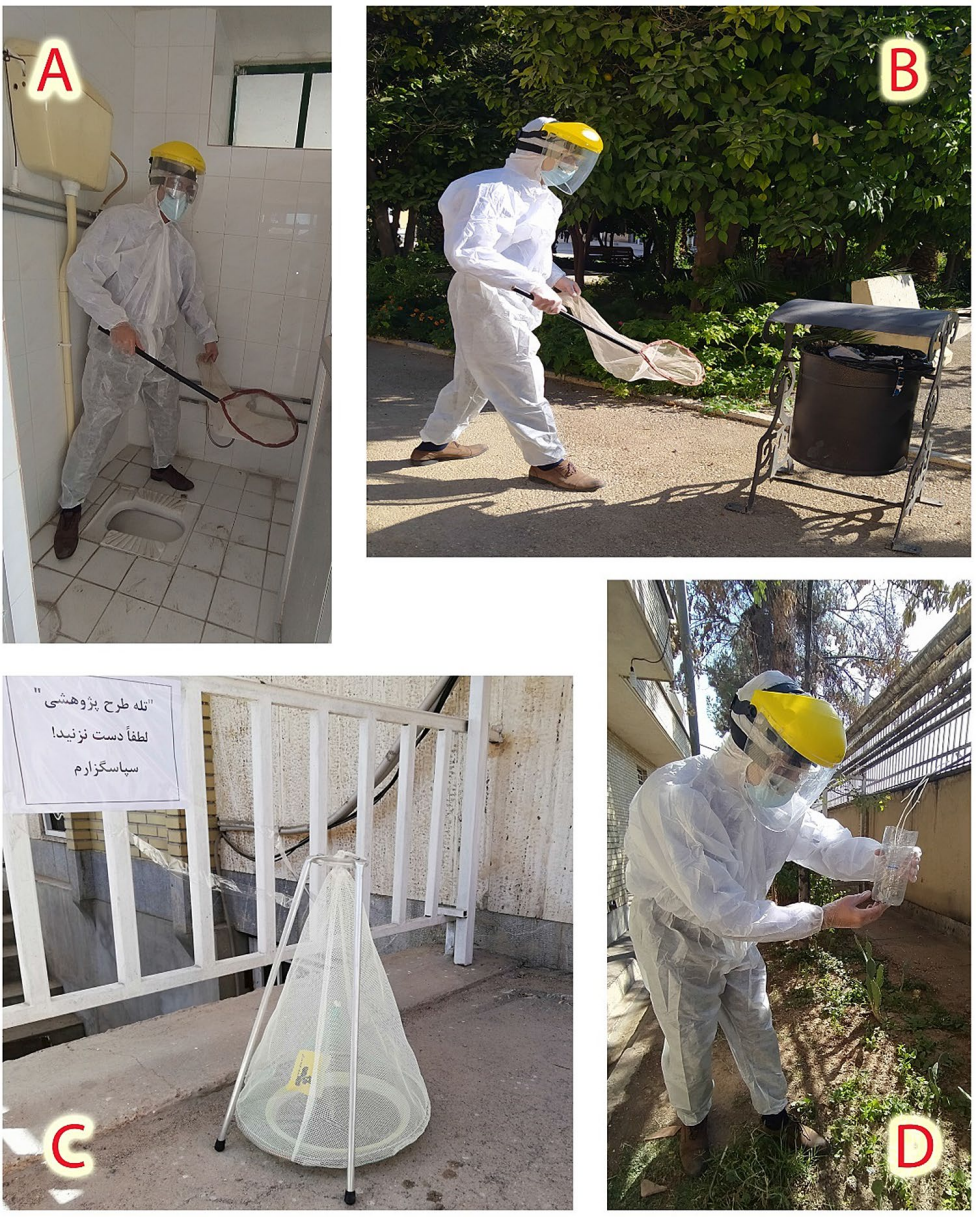
Sampling of adult flies from outdoor using two techniques: (**A**,**B**) net collection in different parts of the hospital grounds, (**C**,**D**) two applied baited traps (standard and man-made types).

Sampling methods including net collection and baited traps (Sabz Avar Co. and a man-made type) were used to catch adult flies (Fig. 2)^19^. After collection, the flies were transferred live to the Medical Entomology Laboratory of Shiraz health school. All causations were made to prevent any secondary viral pollution during sample delivery to the laboratory or sample preparation. All collected specimens were killed by placing them in a freezer and were identified morphologically using valid systematic keys^20–22^. The samples were then transferred to the molecular laboratory under standard conditions for doing further studies to detect and isolate the SARS-CoV-2 from the collected specimens.

### Molecular experiments

#### Samples preparation

To prepare the collected samples, 1 mL of sterile phosphate-buffered saline (PBS; 135 mmol/L NaCl, 3 mmol/L Na_2_HPO_4_·12H_2_O, and 13 mmol/L NaH_2_PO_4_·2H_2_O, pH 7.2) was added for every five mosquitoes placed into 2 mL microtubes to washout the external surface of flies. The supernatant was transferred into a new 1.5 mL collection microtubes (S1). In the next step, the external body of flies was washed by 1 mL sterile PBS followed by 70% ethanol washing. The second PBS supernatant was transferred into a new 1.5 mL collection microtubes (S2). The washed bodies were homogenized together with sterile mortar and pestle while 1 mL of PBS was added gently. The homogenate was transferred to new 1.5 mL collection microtubes and centrifuged at 5000 rpm for 5 min at 4 °C. The collected supernatant (H1), as well as S1 and S2, were stored at − 80 °C until RNA extraction.

#### Nucleic acid extraction

Viral RNA extraction was performed using SinaPure Viral kit (SinaClon BioScience, Iran). 200 µL of each S1, S2, and H1 specimens were subjected to extraction using a column-based kit according to the kit instruction.

#### Taq Man real-time PCR assay

The SARS-CoV-2 test Kit was a molecular in vitro diagnostic technique that uses Taq Man probe-based technology for the qualitative detection of SARS-CoV-2. The "COVID-19 One-Step RT-PCR" kit (COVITECH, Iran) was utilized to perform quantitative real-time PCR tests. The SARS-CoV-2 PCR assay consists of a primer/probe set that amplifies E-gene (labeled with FAM) and S-gene (labeled with ROX) and detects RNaseP (labeled with HEX) gene as an internal control.

A negative (no template) control was used to eliminate the possibility of sample contamination on the assay run. Also, a positive template control was used to verify that the assay run was performing as intended on every tested assay plate. The positive control must be positive at a Ct value of ct:35.

An internal control targeting RNase P was used to verify that nucleic acid was present in every sample and was used for every sample processed. The tests were performed using the StepOnePlus Real-Time instrument (Thermo Fischer Scientific, Applied Biosystems).

### Ethical considerations

All experiments were conducted following the Declaration of Helsinki. This study was approved by the ethics committee of Shiraz University of Medical Sciences (SUMS) and is registered with ID number IR.SUMS.REC.1399.003.

## Results

From the total of 156 adult *Musca domestica* L., forty specimens were considered for detection of the SARS-CoV-2 using Taq Man real-time PCR assay. External body samples, S1 and S2, and homogenate specimens for every 5 flies were utilized to assess for the presence of the virus (Table 1).

**Table 1:** Detection of the SARS-CoV-2 virus using Taq Man real-time PCR assay.

Location	Time of sampling	External body surface-1 samples	Result	External body surface-2 samples	Result	Homogenates	Result
Ali-Asghar hospital	May 11, 2020	S1-1	2000	S2-1	0	H1	100
Chamran hospital		S1-2	700	S2-2	0	H2	0
Ali-Asghar hospital	May 25, 2020	S1-3	200	S2-3	0	H3	0
Chamran hospital		S1-4	0	S2-4	0	H4	0
Ali-Asghar hospital	Jun 8, 2020	S1-4	1500	S2-4	0	H5	200
Chamran hospital		S1-5	250	S2-5	0	H6	100
Ali-Asghar hospital	Jun 22, 2020	S1-7	0	S2-7	0	H7	0
Chamran hospital		S1-8	1000	S2-8	0	H8	0

**Figure 3:**
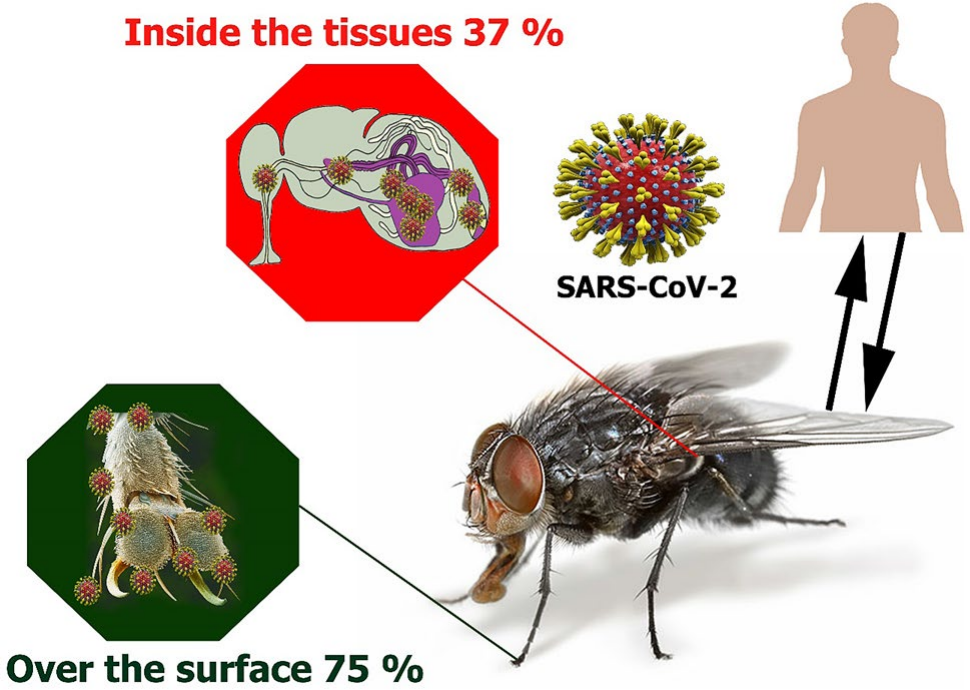
Molecular detection of SARS-CoV-2 in field-collected ‘house flies’.

A Real-time PCR amplification plot for SARS-CoV-2 detection was obtained. From all the investigated flies, 75% of external body samples were positive in terms of contamination to the SARS-CoV-2 virus with a different load of virus (Table 1). All second-washed samples were negative for the SARS-CoV-2. Also, 37% of the homogenized specimens were positive for the new coronavirus, but with less load than body surface samples (Table 1, Fig. 3).

## Discussion

Air droplets and fomites are considered as two main mechanisms for SARS-CoV-2 virus transmission. Insects can also be considered as a new tool for transmitting emerging diseases due to their body structure as well as their high abundance and activity in the environment.

Although the World Health Organization (WHO) has not yet confirmed the role of insects in the transmission of Covid-19 infection, scattered studies have been conducted in various parts of the world on the transmission of viral respiratory infections through insects. These studies have proven the biological and mechanical transmission of some viruses that cause respiratory infections in humans and animals by files^4–14^.

In 2000, the laboratory transmission of turkey Coronavirus was evaluated by a species of beetle called *Alphitobius diaperinus* (Coleoptera: Tenebrionidae). The results showed that turkey coronavirus can be transmitted mechanically, in a limited way, by this insect^13^.

Another study was performed in 2005 to evaluate the possibility of biological and mechanical transmission of the Reticuloendotheliosis virus by two insects (*Culex pipiens* mosquito and *Musca domestica*) in vitro and in the field. The results showed that the mosquitoes that were fed the virus had the virus in their bodies for up to 5 h. Houseflies, on the other hand, survived the virus for up to 72 h. Regarding the samples collected from the field, all samples were negative in terms of virus infection. In general, this study also introduced houseflies as a possible mechanical vector of the virus^23^.

Another study was conducted in 2002 on the housefly (*Musca domestica* L.) and the possibility of transmission of the turkey coronavirus (NC95 strain) strain by this insect. In this study, the larvae of flies were fed with virus-infected food and after their adult release in a turkey breeding environment, it was found that the disease rate was significantly associated with an increase in flies. In this study, flies have been suggested as a potential mechanical transmitter^5^.

In 2010, researchers examined the possible role of houseflies in transmitting Newcastle disease (Paramyxoviridae) in vitro and in the field. In this study, 2000 flies were collected from the field and evaluated for viruses. In the immature laboratory, flies were also exposed to the La Sota strain virus, which showed that the virus could be detected in the gastrointestinal tract of flies for up to 72 h. Although the virus was not isolated in the field samples, flies were suggested as a possible mechanical vector for this virus^24^.

Also, the persistence of Avulavirus in two fly species of *Musca domestica* and *Fannia canicularis* was evaluated. Both species had the infective virus in their bodies for up to one day^6^. Similar studies were conducted in 2007 by researchers in other countries on the transmission of the Newcastle virus by houseflies^14^.

In a 2013 study in Thailand, the possibility of in vitro transmission of the avian influenza virus (HPAI) subtype H5N1 virus was evaluated. In this study, 1500 adult flies were used in three different groups. Group one had no contact with the virus at all, but groups 2 and 3 were exposed to the virus with food for 15 min. Group 2 was immediately killed, homogenized, and given to sensitive birds. Group 3 was homogenized after 24 h. The results showed that all chickens in groups 2 and 3 died, whereas the load of the virus was more in group 2. No mortality was observed in the control group. This study identified houseflies as potential mechanical vectors of this viral disease among birds^12^. Another article in Japan identifies blowflies as a possible and mechanical vector of H5N1 bird flu^11^.

In one of the most recent studies conducted in Iran in 2020, the in vitro acquisition and maintenance of the avian influenza virus (AIV) H9N2 by houseflies was examined. In this study, which was conducted at the laboratory level, adult flies were fed by the virus and the results showed that the virus can survive up to 24 h on the body surface and up to 96 h in internal organs. This study identifies houseflies as a possible and mechanical vector of avian influenza virus^10^.

Recently, the potential of house flies to mechanically transmit SARS-CoV-2 was investigated by Balaraman et al. The results showed that, under laboratory conditions, house flies acquired and harbored SARS-CoV-2 for up to 24 h post-exposure. They mentioned that house flies were able to mechanically transmit SARS-CoV-2 genomic RNA to the surrounding environment up to 24 h^25^.

The role of other blood-sucking insects such as mosquitoes in transmitting the COVID-19 has also been discussed^9^. Some articles have also suggested that flies may be involved in the mechanical transmission of the Covid-19 virus^4,8^.

Based on the WHO report, there is no certainty about the survival duration of the SARS-CoV-2 on surfaces, but this virus seems to act like other coronaviruses. Previous studies show that coronaviruses, including the SARS-CoV-2, can survive on surfaces for 2 h to 9 days. This may vary under different conditions (e.g., type of surfaces, temperature, relative humidity, and characteristics of the virus strain)^8,26,27^.

Therefore, due to the relatively high persistence of the virus, there is a possibility of flies coming into contact with the virus and up taking them through contact and feeding in high-risk environments such as hospitals and their waste disposal sites. In this case, due to the high locomotor activity of these insects and also their synanthropic behavior, the possibility of mechanical transmission of the SARS-CoV-2 will not be far from the mind.

It has been proven that fomites (infected surfaces) play an important role in the spread of viruses, including SARS-CoV-2^2^. Based on the results of this study, the possibility of transmission of the SARS-CoV-2 by houseflies through the fomites is quite probable.

The possibility of binding of SARS-CoV-2 to the insect epithelium surface molecules should be significantly considered. SARS-CoV-2 has a superior binding capacity to the surface sialic acids with the spike protein flat N-terminal domain. Therefore, the virus spike protein is capable of faster interaction with the surface glycans which is likely to be the core mode of action of its faster infection with the lower amount of virion, leading to the pandemic grade contagious capacity^16,28,29^.

Similar entry receptors are available in the insects' surface bodies. C-type lectin receptors were detected from the housefly epithelium surface^30^. Moreover, some glycoproteins have been identified from the epithelium surface of a housefly that could be bound to SARS-CoV-2 molecules^17^. Therefore, the possibility of binding the virus with surface molecules of the housefly is very probable and can strengthen the theory of the mechanical transmission of the SARS-CoV-2 by these vectors.

Since fecal transmission of the virus has been confirmed in patients, fecal feeding and mechanical transmission can play an important role in disease transmission^31^. If the environment is not properly managed, SARS-CoV-2 can be transmitted by mechanical transmission by insects^32^.

In our study, the SARS-CoV-2 was isolated from a high percentage of field-collected flies (75%). Surprisingly, the rate of infection of the external surface of the flies with the virus was high in both surveyed hospitals. This high rate of contamination indicates that these insects with high abundance and mobility are most likely involved in the mechanical transmission of this mysterious virus in crowded and high-risk places such as hospitals and pose a serious threat to human health and preventive measures should be taken to control such insects and their mobility pathways between infection intensive care units and normal parts of a hospital as an example.

In addition, detecting the virus in one-third of homogenized specimens could warn us that flies may be able to support the virus in their bodies and transmit it biologically to humans. However, expressing a detailed opinion on this issue requires additional and more accurate research in the future based on Koch's postulates for the infection of the insects and animal model for the transmission^33^.

Another interesting and warning point obtained in this study was that, in total, the virus load was higher in the samples taken from Ali-Asghar Hospital than in the samples collected from Chamran Hospital. This is because Ali-Asghar Hospital is the main referral center for Covid-19 patients in the province and the number of patients referred to this medical center is much higher.

These results can also confirm that the more people with symptoms as well as asymptomatic carriers are present in the community, the greater load of the virus will be present in the environment. As a result, some active and synanthropic insects such as house flies can play an effective role in the transmission of this disease in the community by infecting the external surface of their body as well as their internal organs.

Perhaps one of the reasons for the transmission of the disease in the warm months of the year can be attributed to the role of these medically important insects in the epidemiological cycle of the disease. Therefore, it is required to consider the role of insects in disease transmission, especially in overcrowded areas with poor health conditions and also high-risk areas (such as hospitals and health care centers).

## Conclusion

Mechanical or biological transmission of the virus by flies can greatly complicate the epidemiology of the diseases^34^. In this case, the role of vector control along with other methods of prevention and control of the disease will be very important. Health policymakers should plan and implement an integrated vector management (IVM) program in high-risk environments during the warm seasons of the year in temperate regions as well as for all months of the year in tropical regions when the population and activity of these vectors increase^18^. Further in vivo studies on the laboratory and field are needed to examine the exact role of these insects in the transmission of this deadly universal virus.

## Data Availability

The data that support the findings of this study are available from the corresponding author, [AS], upon reasonable request.
